# Chemical composition and enzymatic digestibility of sugarcane clones selected for varied lignin content

**DOI:** 10.1186/1754-6834-4-55

**Published:** 2011-12-06

**Authors:** Fernando Masarin, Daniela B Gurpilhares, David CF Baffa, Márcio HP Barbosa, Walter Carvalho, André Ferraz, Adriane MF Milagres

**Affiliations:** 1 Departamento de Biotecnologia, Escola de Engenharia de Lorena, Universidade de São Paulo, CP 116, 12602-810 Lorena, SP, Brasil; 2 Departamento de Fitotecnia, Universidade Federal de Viçosa, Viçosa, MG, Brazil

## Abstract

**Background:**

The recalcitrance of lignocellulosic materials is a major limitation for their conversion into fermentable sugars. Lignin depletion in new cultivars or transgenic plants has been identified as a way to diminish this recalcitrance. In this study, we assessed the success of a sugarcane breeding program in selecting sugarcane plants with low lignin content, and report the chemical composition and agronomic characteristics of eleven experimental hybrids and two reference samples. The enzymatic digestion of untreated and chemically delignified samples was evaluated to advance the performance of the sugarcane residue (bagasse) in cellulosic-ethanol production processes.

**Results:**

The ranges for the percentages of glucan, hemicellulose, lignin, and extractive (based on oven-dry biomass) of the experimental hybrids and reference samples were 38% to 43%, 25% to 32%, 17% to 24%, and 1.6% to 7.5%, respectively. The samples with the smallest amounts of lignin did not produce the largest amounts of total polysaccharides. Instead, a variable increase in the mass of a number of components, including extractives, seemed to compensate for the reduction in lignin content. Hydroxycinnamic acids accounted for a significant part of the aromatic compounds in the samples, with *p*-coumaric acid predominating, whereas ferulic acid was present only in low amounts. Hydroxycinnamic acids with ester linkage to the hemicelluloses varied from 2.3% to 3.6%. The percentage of total hydroxycinnamic acids (including the fraction linked to lignin through ether linkages) varied from 5.0% to 9.2%, and correlated to some extent with the lignin content. These clones released up to 31% of glucose after 72 hours of digestion with commercial cellulases, whereas chemically delignified samples led to cellulose conversion values of more than 80%. However, plants with lower lignin content required less delignification to reach higher efficiencies of cellulose conversion during the enzymatic treatment.

**Conclusion:**

Some of the experimental sugarcane hybrids did have the combined characteristics of high biomass and high sucrose production with low lignin content. Conversion of glucan to glucose by commercial cellulases was increased in the samples with low lignin content. Chemical delignification further increased the cellulose conversion to values of more than 80%. Thus, plants with lower lignin content required less delignification to reach higher efficiencies of cellulose conversion during the enzymatic treatment.

## Background

Sugarcane residue (bagasse) is an abundant agricultural residue and a promising substrate for ethanol production [1]. Although it contains enough cellulose to be an excellent source of sugars for ethanol production, the conversion of cellulose to glucose by enzymes is very limited without the use of an efficient lignocellulose pretreatment. The recalcitrance of lignocellulosic materials is related to several factors, including the close association of cellulose with hemicellulose and lignin in the cell wall, which hinders the cellulase action [2]. One of the ways to increase the cellulose conversion by enzymes in such materials involves lignin degradation or removal by pretreatment [3-6], which increases the cell-wall porosity, facilitating enzyme infiltration and cellulose hydrolysis [7,8].

Some recent work has focused on depleting lignin by breeding new cultivars or transgenic plants as a way to diminish lignocellulose recalcitrance [9-13]. Grabber *et al*. [14] reported that artificially lignified cell walls from maize had their digestibility to ruminal biota decreased as a function of increased lignification, and the authors concluded, on the basis of the use of different lignin precursors, that the engineering of plants for reduced lignification or ferulate-lignin crosslinking improves fiber digestibility to a greater degree than does shifting lignin composition (for example, by selecting high syringyl content in lignified plants). This finding is relevant because in grasses, part of the recalcitrance is associated not only with the occurrence of lignin in cell walls but also with the presence of hydroxycinnamic acids linked primarily to the hemicelluloses [8,14,15]. It is also known that cell-wall digestibility varies significantly among plant varieties [12]. Part of this variation is often associated with the total lignin concentration and the presence of hydroxycinnamic acid crosslinks in the cell walls [12,15].

Recently, a sugarcane breeding program in Brazil, RIDESA (Academic Network for the Development of Sugar-Alcohol Sector; http://www.ridesa.com.br) has been making efforts to select sugarcane plants with low lignin content and/or altered lignin composition [16], using a recurrent selection method to increase the frequency of favorable alleles through repeated cycles of crossing and selection. In this paper, we report the chemical composition and some agronomic characteristics of 11 experimental hybrids. Enzymatic digestion of the untreated sugarcane bagasse from those hybrids and of some chemically delignified samples was evaluated in an attempt to enhance the performance of the sugarcane bagasse in cellulosic-ethanol production programs.

## Results and discussion

### Chemical composition and field productivity of the sugarcane clones

#### Samples

Eleven sugarcane bagasse samples obtained from experimental sugarcane hybrids were evaluated according to their chemical composition. Two reference materials were included in this study: a widely grown sugarcane cultivar (the reference cultivar) and a sugarcane bagasse sample collected from a mill that crushes a mix of commercial sugarcane cultivars (the mill bagasse sample) (Table 1). The bagasse samples varied in their chemical compositions and were ranked according to their lignin content. Roughly, the percentages of glucan, hemicellulose, lignin and extractive content of the samples ranged from 38% to 43%, 25% to 32%, 17% to 24%, and 2% to 7%, respectively. Summative data were in the range 88% to 96%. The chemical 4-O-methyl-glucuronic acid, which is often found as side chains in the hemicellulose of sugarcane bagasse [17], was not quantified in these samples, and might account for part of the 'undetermined compound' content [18]. The acid-soluble ash and sugar-degradation products formed during acid hydrolysis of the samples would also account for a small part of this [18].

**Table 1:** Chemical composition* of sugarcane bagasse samples obtained from experimental sugarcane hybrids ranked† by their lignin content

Clone	Total lignin	Hemicellulose	Glucan	Extractives	Sum
89	16.8 ± 0.1^a^	27.3 ± 0.3^a,c,d,f,i,j,k,l,m^	40.3 ± 0.1^a,b,c,d,f,g,h,i,j,k,l,m^	7.5 ± 0.1^a^	92.0
146	18.6 ± 0.1^b,c,d^	31.6 ± 0.8^b,e,g,h^	40.9 ± 0.3^b,c,d,e,f,g,h,i,j,l^	2.4 ± 0.1^b,c,d,e,g,h,i,l,m^	93.5
58	18.6 ± 0.1^c,d^	26.3 ± 0.1^c,d,f,i,j,k,l,m^	40.9 ± 0.3^c,d,e,f,g,h,i,j,l^	2.6 ± 0.2^c,d,e,h,i,l,m^	88.4
53	19.4 ± 0.5^d,e,f,g^	27.1 ± 0.4^d,f,i,j,k,l,m^	42.2 ± 0.5^d,e,f,g,h,i,l^	2.7 ± 0.1^d,e,h,i,l,m^	91.3
166	19.6 ± 0.5^e,f,g,h,i^	31.5 ± 0.1^e,g,h^	43.2 ± 0.4^e,f,h,l^	1.9 ± 0.1^e,g,h,l,m^	96.2
87	19.7 ± 0.1^f,g,h,i,j^	27.3 ± 0.8^f,i,j,k,l,m^	42.2 ± 0.3^f,g,h,i,l^	3.9 ± 0.4^f,i^	93.1
321	20.2 ± 0.4^g,h,i,j^	31.0 ± 1.0^g,h^	40.4 ± 0.5^g,h,i,j,k,l,m^	1.6 ± 0.3^g,l^	92.9
50	20.5 ± 0.1^h,i,j^	30.0 ± 2.0^h,j^	42.0 ± 1.0^h,i,l^	2.5 ± 0.1^h,i,l,m^	94.6
8	20.5 ± 0.4^i,j^	26.6 ± 0.7^i,j,k,l,m^	40.0 ± 1.0^i,j,k,l,m^	3.2 ± 0.4^i,m^	90.0
121	20.6 ± 0.1^j,k^	28.2 ± 0.5^j,k,l^	39.0 ± 1.0^j,k,m^	4.9 ± 0.2^j,k^	92.9
140	21.5 ± 0.2^k^	27.0 ± 0.3^k,l,m^	38.2 ± 0.5^k,m^	5.1 ± 0.5^k^	91.8
MB	24.0 ± 0.1^l,m^	26.0 ± 1.0^l,m^	42.0 ± 2.0^l^	2.2 ± 0.4^l,m^	93.8
RC	24.5 ± 0.5^m^	25.2 ± 0.4^m^	38.2 ± 0.2^m^	2.6 ± 0.1^m^	90.5

*Data are mean ± standard deviation.

‡Mill bagasse.

§Reference cultivar.

In each column, the values with the same superscript letters do not differ among themselves at significance level of 0.05.

#### Hemicellulose content

The hemicellulose content was calculated on the basis of the monomeric sugars and acetic acid released after acid hydrolysis. Under the analytical conditions used, xylose, mannose, and galactose eluted at the same retention time, and appeared as a single peak. To assess the levels of these individual sugars in the sugarcane samples, some of the experimental clones and the mill bagasse were analyzed using a pulsed amperometric detector [19]. The chromatograms showed no detectable mannose and galactose in the mill bagasse sample, whereas small peaks of galactose were detected in clones 87, 89, and 140, corresponding, respectively, to 0.90%, 0.83%, and 0.71% of the oven-dry mass of the plant material. These data suggest that the hemicellulose content detected in the evaluated sugarcane samples consisted mainly of xylan backbones ramified with arabinose and acetic acid. The hemicellulose probably contains 4-O-methyl-glucuronic acid also, as this is well documented in the literature for hemicelluloses from sugarcane [17,20]. The molar ratios of xylose and arabinose were similar for all samples, whereas acetic acid varied slightly, giving a substitution pattern of 10 xylose to 1 arabinose to 3 or 4 acetic acid for the xylan structures. This pattern of branching in the structure of xylan in sugarcane is in the same range as previously reported data [17,20].

#### Extractives content

The content of extractives also varied significantly between the samples (Table 1). Bagasse from commercial sugarcane varieties generally contains ethanol-soluble extractives in the range of 1.5% to 3.0% [21,22]. We found results within this range for the mill bagasse and the reference cultivar (2.2% and 2.6%, respectively), but some of the clones contained very high levels of extractives (for example, 5.1% and 7.5% in clones 140 and 89, respectively). The ethanol-soluble fraction is characterized by the presence of waxy materials and low molar mass aromatics, but extraction with 95% ethanol can also dissolve small amounts of oligosaccharides present in the sugarcane samples [23].

To allow discrimination between the classes of compounds present in the extractives fraction, seven samples were selected for detailed evaluation of the extractives. The selected samples comprised the two reference samples and five clones with high, intermediate and low levels of extractives (Table 2). The fraction extracted with 95% ethanol was assessed for the concentrations of aromatics and total carbohydrate, and the remaining material was considered an estimate of the waxy extractives. The data indicated that carbohydrates comprised only small amounts of the extractives (Table 2), whereas the aromatic fraction made up approximately half of the extractives fraction for most of the samples, with a clear exception being clone 89, in which aromatics comprised only 14% of the total extractives. In this clone, an oily extractive was present after ethanol evaporation, which corroborates the predominance of waxy extractives in this clone. This clone also contained the highest content of extractives overall.

**Table 2:** Chemical characteristics of the fraction extracted with 95% ethanol from sugarcane bagasse samples

Sample	Total extractives, %	Compounds, mg/g		
		
		Aromatics	Carbohydrates	Waxes*
89	7.5	144	86	770
166	1.9	405	88	507
87	3.9	434	59	507
321	1.6	382	35	583
140	5.1	377	59	564
MB†	2.2	609	186	205
RCr‡	2.6	382	74	544

*Calculated by subtracting the amounts of aromatics and sugars from the total amount of extractives.

†Mill bagasse.

‡Reference cultivar.

#### Lignin content

Comparison of the entire dataset (Table 1) using one-way analysis of variance showed that the lignin content differed between samples, as follows: 1) the reference cultivar and mill bagasse had the highest lignin content (these samples did not overlap with the clone containing the highest lignin content, which was clone 140); 2) clone 89 had the lowest lignin content (differing significantly from all other samples); and 3) clones 58 and 146 had the second lowest lignin content but they overlapped with clone 53 (which overlapped with the next lowest. There was more overlap for the polysaccharide content, thus the samples could not be classified into groups on this basis. However, the extractives content could also be classified, as follows: 1) clone 89 had the highest extractives content; 2) clones 121 and 140 had the second highest extractives content but differed from clone 89; and 3) most of the clones had extractives content ranging from 1.6% to 3.9%.

#### Polysaccharide levels

The overall assessment of the chemical composition data showed that the samples with the smallest amounts of lignin did not contain the largest amounts of total polysaccharides (Table 1). This lack of correlation suggests that hybrids depleted in lignin do not have a corresponding increase in the amount of a single major component (such as polysaccharides); instead, there seems to be a variable increase in the mass of a number of components, including extractives, to compensate for the reduction in lignin content. These results differ from previous results obtained with transgenic alfalfa, which showed that plants with the lowest lignin content had the highest total carbohydrate levels [9].

#### Hydroxycinnamic acid content

Hydroxycinnamic acids account for a significant percentage of the aromatic compounds found in grasses [15,24,25]. Even total lignin, as determined by the Klason procedure, contains significant amounts of these compounds, owing to condensation reactions that occur during acid hydrolysis [8,25]. In this study, we evaluated the hydroxycinnamic acid content in the samples (Table 3). Part of the total hydroxycinnamic acid content found in sugarcane can be released by a mild alkaline treatment. This portion corresponds mainly to hydroxycinnamic acids that are ester-linked to methylglucurono-arabino-xylans, but that are not etherified to the lignin moieties. The remaining hydroxycinnamic acids are linked to lignin through ether linkages involving the phenolic oxygen of the hydroxycinnamic acid [24]. Our data show that *p*-coumaric acid predominated in all the samples, whereas ferulic acid was detected at lower levels (Table 3). These data agree with some previous characterization results for sugarcane samples [26], but contrast with those from other grasses such as *Phalaris aquatica*, *Lolium perenne*, *Avena saliva *and *Triticum aestivum*, in which ferulic acid is the predominating hydroxycinnamic acid [15,24,27]. The presence of ferulic and *p*-coumaric acids in the alkaline extracts was confirmed by GC/MS analysis. HPLC analysis was unable to detect sinapic acid, but small amounts were detected by the GC/MS analysis.

**Table 3:** Hydroxycinnamic acid composition of sugarcane bagasse samples obtained from experimental sugarcane hybrids ranked by their lignin content

Clone	Hydroxycinnamic acids, g/100g of untreated bagasse					
	
	Released by mild alkali treatment			Released by severe alkali treatment		
	
	Ferulic	Coumaric	Sum	Ferulic	Coumaric	Sum
89	0.50 ± 0.01^a,b,c,d,f,h,i,j,k,l^	1.8 ± 0.1^a,d,f,h,l^	2.3 ± 0.1^a,d,f,l^	1.1 ± 0.1^a,b,c,d,e,f,g,h,i,j,k,l^	3.9 ± 0.5^a,c,d,f,h,j,k^	5.0 ± 0.5^a,c,d,f,h,i,j,k^
146	0.59 ± 0.02^b,c,e,g,h,i,j,k,m^	2.5 ± 0.2^b,c,d,e,f,g,h,i,j,k,l,m^	3.1 ± 0.2^b,c,d,e,f,g,h,i,j,k,m^	1.4 ± 0.1^b,e,f,g,h,l,m^	6.2 ± 0.5^b,c,d,e,f,g,h,i,j,k,l,m^	7.6 ± 0.5^b,e,f,g,h,i,j,k,l,m^
58	0.59 ± 0.01^c,e,g,h,i,j,k,m^	2.6 ± 0.1^c,d,e,f,g,h,i,j,k,m^	3.2 ± 0.1^c,d,e,g,h,i,j,k,m^	0.8 ± 0.2^c,d,f,i,j,k^	4.4 ± 1.2^c,d,e,f,h,i,j,k,l^	5.2 ± 1.2^c,d,f,h,i,j,k^
53	0.49 ± 0.02^d,f,h,i,j,k,l^	2.2 ± 0.1^d,e,f,h,i,j,k,l,m^	2.7 ± 0.1^d,f,h,i,j,k,l^	0.9 ± 0.1^d,f,h,i,j,k^	4.6 ± 0.9^d,e,f,h,i,j,k,l^	5.5 ± 0.9^d,f,h,i,j,k,l^
166	0.63 ± 0.05^e,g,h,i,m^	2.8 ± 0.3^e,g,h,i,j,k,m^	3.4 ± 0.3^e,g,h,i,j,k,m^	1.4 ± 0.1^e,f,g,h,l,m^	6.2 ± 0.2^e,f,g,h,i,j,k,l,m^	7.6 ± 0.2^e,f,g,h,i,j,k,l,m^
87	0.42 ± 0.04^f,j,l^	2.1 ± 0.3^f,h,i,k,l^	2.5 ± 0.3^f,h,k,l^	1.1 ± 0.1^f,g,h,i,j,k,l^	5.7 ± 0.9^f,g,h,i,j,k,l,m^	6.8 ± 0.9^f,g,h,i,j,k,l^
321	0.65 ± 0.01^g,m^	2.9 ± 0.1^g,h,i,j,k,m^	3.6 ± 0.1^g,h,i,j,k,m^	1.4 ± 0.1^g,h,l,m^	6.8 ± 0.5^g,h,i,j,k,l,m^	8.2 ± 0.5^g,h,i,j,k,l,m^
50	0.55 ± 0.03^h,i,j,k,l,m^	2.4 ± 0.2^h,i,j,k,l,m^	3.0 ± 0.2^h,i,j,k,l,m^	1.2 ± 0.1^h,i,j,k,l^	5.2 ± 0.4^h,i,j,k,l^	6.4 ± 0.4^h,i,j,k,l^
8	0.55 ± 0.07^i,j,k,l,m^	2.7 ± 0.4^i,j,k,m^	3.3 ± 0.4^i,j,k,m^	1.0 ± 0.1^i,j,k^	6.0 ± 0.2^i,j,k,l,m^	7.0 ± 0.2^i,j,k,l^
121	0.50 ± 0.04^j,k,l^	2.8 ± 0.3^j,k,m^	3.3 ± 0.3^j,k,m^	0.9 ± 0.1^j,k^	5.8 ± 0.5^j,k,l,m^	6.7 ± 0.5^j,k,l^
140	0.53 ± 0.02^k,l,m^	2.6 ± 0.1^k,m^	3.0 ± 0.1^k,l,m^	1.0 ± 0.1^k^	5.2 ± 0.3^k,l^	6.2 ± 0.3^k,l^
MB‡	0.47 ± 0.01^l^	1.9 ± 0.1^l^	2.4 ± 0.1^l^	1.4 ± 0.2^l,m^	6.1 ± 0.8^l,m^	7.5 ± 0.8^l,m^
RC§	0.62 ± 0.01^m^	2.8 ± 0.1^m^	3.4 ± 0.1^m^	1.7 ± 0.2^m^	7.5 ± 1.0^m^	9.2 ± 1.0^m^

*Data are mean ± standard deviation.

†. The same as the Table 1 footnote

‡Mill bagasse.

§Reference cultivar.

**Figure 1:**
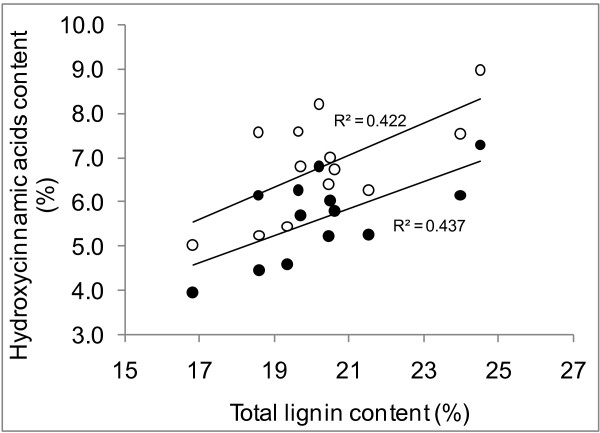
**Correlation of lignin and hydroxycinnamic acid content**. Correlation of lignin and hydroxycinnamic acid content (released by severe alkaline treatment) in sugarcane samples. Black circles denote *p*-coumaric acid content only; white circles denote total hydroxycinnamic acid content.

In the clones analyzed, the percentage of hydroxycinnamic acids ester-linked to the hemicelluloses but containing a free phenolic position (released by the mild alkaline treatment) varied from 2.3% to 3.6%, and were not proportional to the hemicellulose or lignin content of the samples (r^2 ^values for linear correlations were lower than 0.26). By contrast, the content of total hydroxycinnamic acids (released by the severe alkaline treatment) correlated to some extent with lignin (Figure 1). Linear models correlating these variables had r^2 ^values of 0.42 for datasets containing ferulic plus *p*-coumaric acids and 0.44 for those containing *p*-coumaric acid only. This proportionality was expected, as part of the hydroxycinnamic acid content contributes to the total lignin content detected using the Klason analytical procedure. This was also evidenced by the practically constant ratio of hydroxycinnamic acids to lignin, which was between 0.3 and 0.4 for all the samples.

The clones were ranked as a function of the total lignin content (Table 1) because this work focused on the evaluation of sugarcane hybrids as candidate feedstock for cellulosic-ethanol production. It is well documented that lignin acts as a barrier for enzyme infiltration in lignified cell walls, and that it unproductively binds the cellulases during enzymatic conversion of lignocellulosic substrates [2,5,8]. In principle, plants with low lignin content are easier to pre-treat and to hydrolyze by cellulases compared with plants with high lignin content [2,6,9,28].

#### Relationship between plant, biomass and sucrose production with lignin content

Although low lignin content is desirable in plants used for cellulosic-ethanol production, biomass productivity is also relevant. In the particular case of sugarcane, sucrose yield is also a subject of interest [1]. The analyzed productivity parameters indicated that two of the clones combined high plant, biomass and sucrose productivity with low lignin content (clone 58 and 89) (Table 4). Clone 87 had high plant, biomass and sucrose production, with an intermediate lignin content, whereas clone 8 was also in the high productivity range, but was in the group that contained the highest lignin content. By contrast, despite having a low lignin content, clones 53, 146, and 166 had very low plant productivity. Commercial varieties of sugarcane usually contain high levels of lignin, as seen in the varieties evaluated in this work (Table 1). The productivity of commercial plantations varies considerably, depending on the sugarcane variety, the soil and climate conditions, and the harvest schedule [16,29]. In commercial plantations, it is usual to have five succeeding annual harvests before plant replacement. In general, the first of these harvests is usually the most productive, and the productivity decreases after each annual cycle [16]. Crude productivity data for Brazilian plantations can be assumed to vary from 60 to 90 wet tons of sugarcane per hectare, yielding 130 and 135 kg of dry bagasse and sucrose per ton of wet sugarcane, respectively [1,16,29]. Most of the hybrids evaluated in this work had productivity values that were higher than these average values, with the exception of hybrids 146, 166 and 321 (Table 4).

**Table 4:** Plant productivity parameters, biomass content and sucrose yield of experimental sugarcane hybrids ranked by their lignin content

Hybrid number	Plant productivity, wet ton/hectare	Diameter at internode, mm	Plant bending score*	Dry biomass content (bagasse), kg/ton of wet plant	Sucrose yield, kg/ton of wet plant
89	101.2	25	2	176.0	142.5
146	52.6	20	4	84.4	147.4
58	84.9	32	3	142.4	131.1
53	63.9	25	5	135.4	124.5
166	50.5	28	1	112.4	138.9
87	81.8	25	-	190.2	137.8
321	54.4	30	2	115.2	119.1
50	72.9	24	1	97.4	143.4
8	84.7	25	1	131.2	152.0
121	91.4	26	3	114.8	117.1
140	88.0	33	3	175.8	123.1

*Degree of plant bending in the stalk was scored from 1 (straight) to 5 (bent).

Plant bending is another relevant characteristic in sugarcane, because a high degree of bending makes either manual or mechanized cutting difficult. In general, the data suggest that a low lignin content in the plants favors plant bending during growth, with a clear exception being clone 89, which displayed moderate (level 2) plant bending even though it contained a very low lignin concentration.

### Enzymatic hydrolysis of the sugarcane samples

**Figure 2:**
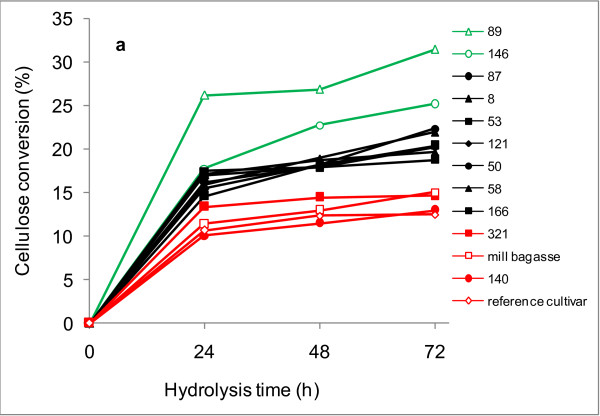
**Cellulose conversion of bagasse**. Conversion of cellulose to glucose by direct enzymatic hydrolysis of sugarcane bagasse samples obtained from experimental sugarcane hybrids varying in lignin content. Average standard deviations, calculated from triplicates, were 9.0%, 6.6% and 5.9% of the experimental values at the reaction times of 24, 48 and 72 hours, respectively.

Direct enzymatic hydrolysis of lignocellulosic materials can provide an indication of their digestibility and suitability for the processes designed for preparing cellulosic ethanol [9,12,14,15]. Therefore, the clones evaluated in this work were hydrolyzed by a mixture of commercial cellulases and hemicellulases. In most cases, the cellulose conversion was found to be less than 20% (Figure 2), which is in accordance with the low digestibility of non-pretreated lignified cell walls [2].

**Figure 3:**
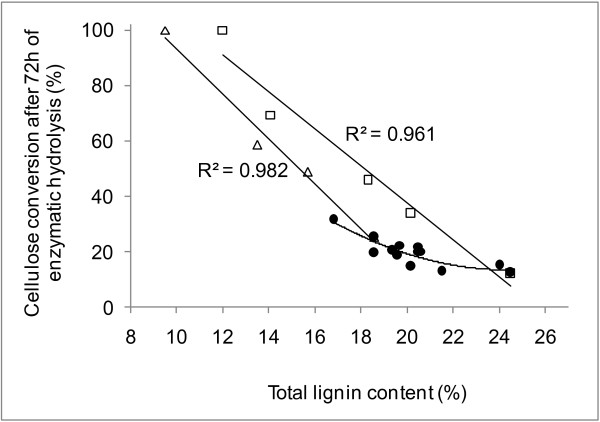
**Cellulose conversion as a function of lignin**. Relationship of cellulose conversion to the lignin content of sugarcane samples. Black circles denote untreated sugarcane clones; white squares denote chlorite-delignified reference sugarcane cultivar; white triangles denote chlorite-delignified experimental hybrid number 146. Average standard deviations, calculated from triplicates, were 5.9%, 5.3% and 3.3% of the experimental values for the untreated samples, chlorite-delignified reference cultivar samples, and chlorite-delignified hybrid 146 samples, respectively.

Three main data groups could be distinguished on the basis of cellulose conversion levels (Figure 2). Of the four samples with the lowest cellulose conversions, three (clone 140, the mill bagasse and the reference cultivar) also had high lignin content (Table 1). The exception was clone 321, which, despite having intermediate lignin content (20.2%), exhibited low levels of cellulose conversion. The largest number of samples (Figure 2, black lines) exhibited cellulose conversion levels of 19% to 22% after 72 hours of enzymatic hydrolysis. In this group, the lignin content was in the intermediate range (Table 1). Clones 89 and 146 could be grouped separately from the others, as they yielded the highest cellulose conversion levels (31% and 25%, respectively) after 72 hours of enzymatic treatment. Interestingly, both clones contained low lignin levels (Table 1). When cellulose conversion levels were plotted against lignin content (Figure 3), a second-order polynomial equation fitted well to the results, with an r^2 ^value of 0.75. The content of other components such as extractives, hydroxycinnamic acids, cellulose or hemicellulose could not be used as predictors of the cellulose conversion because there was a wide distribution in all cases (data not shown). Xylose was also released from the sugarcane bagasse samples during enzymatic digestion, owing to the xylanolytic activities present in the commercial cellulases [6,30]. However, conversion of xylan to xylose was less than 15%, with no apparent differences between the clones (data not shown).

The digestibility data of the sugarcane samples showed that the enzymatic hydrolysis of cellulose increased in clones with diminished lignin content. However, the overall efficiency of the process was still low (31% after 72 hours of hydrolysis) for the clone with the lowest lignin content (16.8%). To challenge the effect of lignin depletion through breeding with lignin depletion by chemical removal from the cell walls, two of the studied samples were further delignified by a selective chemical process using sodium chlorite under acidic pH. Several levels of lignin removal were obtained, depending on the reaction time (Figure 3). This selective chemical delignification produced samples in which the cellulose conversion was significantly enhanced. There was an almost linear correlation between the cellulose conversion levels after enzymatic hydrolysis and the lignin content of these samples. Considering that the slope for the curve obtained with data from clone 146 was higher than that for the reference cultivar (slopes of 8.1 and 6.7, respectively), it is clear that plants bred to contain less lignin will require less delignification to reach a defined level of cellulose hydrolysis. For example, to reach an 80% level of cellulose hydrolysis, clone 146 and the reference cultivar required delignification levels of 37% and 44%, respectively.

The data for enzymatic hydrolysis of the untreated and delignified samples showed that in delignified samples, lignin removal enhances enzymatic digestibility not only because the pretreated material has a lower lignin content, but also because the chemical pretreatment should increase the porosity of the cell walls and the reactivity of the substrates, thus facilitating the hydrolysis of the constituent polysaccharides, as previously reported [8,31]. This finding is in close agreement with previous studies on the evaluation of the recalcitrance in untreated and chemically pretreated lignocellulosic materials [2,5,8,31].

## Conclusions

The current evaluation of 11 sugarcane experimental hybrids selected for varied lignin content showed that some plants had the combined characteristics of high biomass and sucrose productivity with low lignin content. However, the samples with the smallest amounts of lignin did not produce the largest amounts of total polysaccharides. Instead, there was a variable increase in the mass of a number of components, including the group of extractives, which seems to reflect compensation for the reduction in the lignin content.

After enzymatic digestion of the bagasse samples, the cellulose conversion levels increased in the samples with lower lignin content. In non-pretreated samples, the cellulose conversion reached a maximum of 31% after 72 hours of hydrolysis for the clone with the lowest lignin content, 16.8%. By contrast, chemically delignified samples led to cellulose conversion values of more than 80%. Interestingly, plants with lower lignin levels required lower levels of delignification to reach higher efficiencies of cellulose conversion during the enzymatic treatment.

## Methods

### Raw material and biomass preparation

Eleven experimental sugarcane hybrids were selected from the breeding program developed by RIDESA associated with the Federal University of Viçosa, Viçosa, MG, Brazil [16]. Seeds obtained after hybridization were planted in trays and maintained in a greenhouse for 30 days. The seedlings were first transferred to tubs and then to the field to generate initiating plants for subsequent vegetative propagation. The clone plantation was set in May 2007 using rows 5 m in length in an experimental field in Oratórios, MG, Brazil (20°25'50'' latitude south, 42°48'20" longitude west).

The first clonal crop was obtained in July 2008. The plant material that regrew after the first cut (second clonal crop) was harvested in July 2009 (12-month-old plants) and used for the evaluation reported in this paper. Ten stalks taken randomly from each row were used for estimating field-productivity parameters. Plant productivity was estimated from the total number of stalks per row and the wet weight of 10 stalks. Stalk diameter was measured at the fifth internode from the plant base. Plant bending was estimated from a five-point approximate scale varying from 1 (straight; less than 5° of the angle between stalks) to 5 (bent; more than 150° of the angle between stalks). Dry biomass values were obtained after juice extraction of 500 g samples crushed in a hydraulic press. Sucrose yield was determined from polarimetric determination of sugars in the extracted juice [32].

In addition to the 11 experimental hybrids, two reference crops were included in the study. The first was a widely grown sugarcane cultivar often planted in family farms in the state of São Paulo to produce sugar syrup with a high sucrose concentration. This sample included 18-month-old stalks harvested in a farm located in Lorena, SP, Brazil (22°43'51" latitude south, 45°07'29" longitude west), and was termed the 'reference cultivar'. The second reference material was a sugarcane bagasse sample from a sugar and ethanol mill that crushes a mix of commercial sugarcane cultivars. This material, termed the 'mill bagasse' sample, was collected at the mill as freshly crushed material and air-dried to a final humidity of 12%, then stored in plastic bags until used.

For chemical characterization and enzymatic hydrolysis studies, approximately 15 stalks of the harvested hybrids or the reference cultivar were cut by a reaper machine into 5 to 10 mm long fragments. The cut material was blended in water and washed to remove sucrose. To avoid loss of fine particles (< 0.2 mm in length), the material was washed inside a PVC column 1 m long and 150 mm in diameter, with a 200-mesh screen at the bottom. Any particles passing through the screen were pumped back to the top of the column. Filtrate recirculation permitted the formation of a fiber mat at the column base that retained these particles. Water recirculation was stopped when the washing water was clear. After this point, additional blended biomass was applied to the column, and fresh water was passed through the column until the wash produced a negative color result in a phenol/sulfuric acid assay. The obtained biomass material was stored at -18°C until use. For the mill bagasse, the sample biomass was washed with water, air-dried, and stored at room temperature until use.

The reference cultivar and hybrid sample 146 were delignified with a sodium chlorite/acetic acid aqueous solution to evaluate the effect of selective lignin removal on the sample performance under enzymatic hydrolysis. Samples were milled to pass through a 0.84-mm screen, and delignified for reaction periods from 0.25 to 4 hours as described previously [8] to produce samples with progressively decreased lignin content.

### Chemical composition of the samples

Ethanol-soluble extractives were determined by extraction with 95% (v/v) ethanol in a Soxhlet apparatus. The samples were air-dried and milled to pass through a 0.84-mm screen. Approximately 3 g of the milled sample was extracted with 95% ethanol for 6 hours in a Soxhlet apparatus. The percentage of extractives was determined on the basis of the dry weights of the extracted and non-extracted milled samples (data are provided as mean ± standard deviation (SD)). This procedure was conducted in triplicate. Some of the ethanolic extracts were dried under vacuum in a rotary evaporator at 60°C. The obtained solids were then further dried to a constant weight at 60°C, and maintained under vacuum over phosphorus pentoxide in a desiccator. The level of aromatic compounds in these solids was estimated by UV spectroscopy. The solids were suspended in 0.5 mol/l sodium hydroxide at a concentration of approximately 1 mg/ml, and filtered through 0.45 μm membranes, then the absorbance of the resulting solutions was measured at 280 nm. The concentration of aromatic compounds in this solution was estimated to have an average absorptivity of 20 l/cm.g [33]. The total sugar content in the same solution was determined by the sulfuric acid/phenol method using sucrose as the calibration standard [34].

Ethanol-extracted or chlorite-delignified milled samples were hydrolyzed with 72% (w/w) sulfuric acid at 30°C for 1 hour (3 ml of acid to 300 mg of sample) as described previously [35]. The acid hydrolysate was diluted with 79 ml of water, and the mixture was autoclaved at 121°C for 1 hour. The residual material was cooled, and filtered through a porous glass filter (number 3). Solids were dried to a constant weight at 105°C, and assessed as the insoluble lignin component. The concentration of soluble lignin in the aqueous fraction was determined by measuring the absorbance of the solute at 205 nm, using the value of 105 L/cm.g as the absorptivity of soluble lignin [36]. The concentrations of monomeric sugars in the soluble fraction were determined by HPLC (HPX87H column; Bio-Rad, Hercules, CA, USA) at 45°C and an elution rate of 0.6 mL/min with 5 mmol/l sulfuric acid. Sugars were detected in a temperature-controlled refractive index detector at 35°C. Under these conditions, xylose, mannose and galactose eluted at the same time, and appeared as a single peak. Glucose, xylose, arabinose and acetic acid were used as external calibration standards. No corrections were performed because of the sugar-degradation reactions that take place during acid hydrolysis [35]. The factors used to convert sugar monomers to anhydromonomers were 0.90 for glucose and 0.88 for xylose and arabinose. Acetyl content was calculated as the acetic acid content multiplied by 0.72. This procedure was conducted in duplicate (data shown as mean ± SD). Glucose was reported as glucan after correction by the hydrolysis factor, and the other sugars and acetic acid were used to calculate the hemicellulose content.

Some of the acid hydrolysates were also analyzed with a pulsed amperometric detector (871 Advanced BioScan, Metrohm AG, Herisau, Switzerland) to detect hemicellulose sugars as separate peaks, using an analytical column (Carbopac PA10; Dionex Co., Chelmsford, MA, USA) with an elution rate at 1.0 mL/min using a mixture of two eluents: NaOH 50 mmol/l and deionized water. The solvents were mixed automatically to give a ratio of 10% NaOH to 90% H_2_O during the first 20 minutes, then 45% NaOH to 55% H_2_O for 1 minute, followed by 90% NaOH to 10% H_2_O for 9 minutes. Finally, the solvent mixture was set to the initial composition (10% NaOH to 90% H_2_O) for 10 minutes to wash out the system [19].

### Hydroxycinnamic acid extraction and determination

Hydroxycinnamic acids were determined after extraction by alkaline solutions under mild or severe reaction conditions. Mild reaction conditions release hydroxycinnamic acids that are ester-linked to hemicelluloses, whereas severe conditions release the total amount of hydroxycinnamic acids, either ester-linked or ether-linked to the lignocellulose components [25].

Mild reactions were performed with 50 mg of the biomass sample and 5 ml of 1 mol/l NaOH. The sample was incubated at 30°C for 24 hours, using rotary agitation at 120 rpm, then the reaction mixture was acidified to pH 2 with 6 mol/l HCl, and water was added to give a final volume of 10 ml. The mixture was then stored at 4°C for 16 hours, filtered through a 0.45 μm membrane, and analyzed for *p*-coumaric (3-(4-hydroxyphenyl)prop-2-enoic acid) and ferulic (3-(4-hydroxy-3-methoxy-phenyl)prop-2-enoic acid) acids by HPLC. Severe reaction conditions were conducted with 200 mg of the biomass sample and 2 mg of anthraquinone with 16 ml of 4 mol/l NaOH inside 100 mL stainless-steel reactors heated to 170°C for 2 hours [25]. After cooling the reactors, the reaction mixture was acidified to pH 2 with 6 mol/l HCl, and water was added to give a final volume of 100 ml. The mixture was stored at 4°C for 16 hours, and treated as described for the mild-condition samples.

Filtrates of both reaction conditions were analyzed by chromatography (AKTA10 chromatograph; Amersham Biosciences, Piscataway, NJ, USA) equipped with a column 250 mm in length and 4 mm outside diameter (Hypersil; Thermo-Scientific) eluted at 0.8 mL/min with acetonitrile:water (1:4) containing 1% (v/v) of acetic acid. Hydroxycinnamic acids were detected in line at 315 nm.

External calibration was performed using authenticated *p*-coumaric and ferulic acid standards. To correct for partial degradation of hydroxycinnamic acids during the reactions, two calibration curves were built using the same reaction procedures as previously described. Hydroxycinnamic acids were not decomposed under mild reaction conditions. However, severe reaction conditions resulted in 55% and 47% decomposition of ferulic and *p*-coumaric acids, respectively. To determine these degradation levels, authenticated standards were initially dissolved in diethyl ether and quantitatively transferred to stainless-steel reactors filled with 200 mg of filter paper. After the diethyl ether was evaporated, the filter paper-adsorbed compounds were treated under identical reaction conditions as previously described, and the soluble fractions were analyzed by HPLC.

Samples of the reaction mixtures were also evaluated by GC/MS analysis to confirm the presence of the hydroxycinnamic acids. For this evaluation, 4 ml of the acidified mixture was filtered and extracted with an equal volume of ethyl ether (three successive extractions). The ether extracts were combined and dried over anhydrous Na_2_SO_4_, then concentrated under vacuum, and dissolved in 100 μl of pyridine. This solution was left to react with 100 μl of BSTFA (N,O-*bis*-(trimethylsilyl)-trifluoroacetamide) at 60°C for 1 hour [33]. After silylation, the solution was analyzed in an ion trap mass gas chromatograph (Finnigan MAT-GCQ; Thermo Fisher Scientific Inc., Rockford, IL, USA) equipped with a column 30 m long, with inside diameter 0.25 mm and a 0.25 μm film (BPX-5MS; SGE International, Melbourne, VIC, Australia). Column temperature was initially maintained at 80°C for 2 minutes and then heated to 280°C at 10°C/min. This final temperature was maintained for 15 minutes. Helium at 33.0 cm/s was used as the carrier gas. Injector and transfer line temperatures were 240°C and 275°C, respectively. Samples (1 μl) were injected using a split of 1:30. Retention time in the GC and the mass spectrum of each identified peak were identical to that of silylated authenticated standards. The compounds, their retention time (RT, shown in brackets) and main mass spectrum information (m/z, relative intensity) were as follows: *p*-coumaric acid (RT 17.56 minutes), 308 (61; M+), 293 (100), 249 (46), 219 (18), 73 (8); ferulic acid (19.05), 338 (100; M+), 323 (85), 308 (66), 293 (47), 249 (18), 219 (9), 73 (3); sinapic acid (3-(4-hydroxy-3,5-dimethoxyphenyl) prop-2-enoic acid) (20.43), 368 (91; M+), 353 (54), 338 (100), 323 (42), 279 (14), 209 (6), 73 (1).

### Enzymatic hydrolysis of the sugarcane samples

Enzymatic hydrolysis experiments were performed using a mixture of commercial enzyme preparations (Celluclast and Novozym 188; Novozymes, Denmark) at a dosage of 20 FPU plus 40 IU of β-glucosidase per gram of bagasse (oven-dry weight). Each hydrolysis experiment was conducted in 125-ml Erlenmeyer flasks containing 1 g of milled sample (oven-dry weight) and 10 ml of 50 mmol/l sodium-acetate buffer pH 4.8 in addition to the enzyme solution. The flasks were incubated at 45°C with rotary agitation at 120 rpm. The reaction was stopped at defined periods from 24 to 72 hours by heating the flask to 100°C for 5 minutes, followed by centrifugation of the material at 7800 *g *for 15 minutes. The soluble fractions were assayed for glucose and xylose by HPLC using a column (HPX87H; Bio-Rad) at 45°C, with an elution rate of 0.6 mL/min with 5 mmol/l sulfuric acid. Sugars were detected using a temperature-controlled infrared detector set at 35°C. The cellulose and xylan conversion levels reported in the text refer to the conversion of the polysaccharides to their monomers. Values (mean ± SD) for the hydrolysis of the untreated sugarcane samples were estimated from triplicate runs performed with the reference cultivar and experimental hybrid number 58, 140, and 146. The SD values for the hydrolysis experiments of the chlorite-delignified samples were estimated from triplicate runs for each evaluated sample.

## List of abbreviations

FPU: filter paper unit; GC/MS: gas chromatography/mass spectrophotometry; HPLC: high-performance liquid chromatography; PVC: polyvinyl chloride; UV: ultraviolet.

## Competing interests

The authors declare that they have no competing interests.

## Authors' contributions

FM and DG performed chemical analyses, delignification experiments, enzymatic hydrolysis of samples and data interpretation. DCFB and MB provided the experimental sugarcane hybrids, performed field trials, determined the agronomic productivity data, data interpretation and reviewed the manuscript. WC participated in the design of the study, data interpretation and reviewed the manuscript. AF and AMFM conceived the study, participated in chemical analyses and enzymatic digestion studies, data interpretation and reviewed the manuscript. All authors read and approved the final manuscript.
